# Supplementary material for: Using Convolutional Neural Networks for the Classification of Suboptimal Chest Radiographs
Source: J Med Radiat Sci. 2025 Jul 18;73(1):60–8. doi: 10.1002/jmrs.70006 (PMC12950515; doi:10.1002/jmrs.70006)
Supplement: Supplementary file 1 — Appendix S1. [file JMRS-73-60-s001.docx]

**Supporting information**


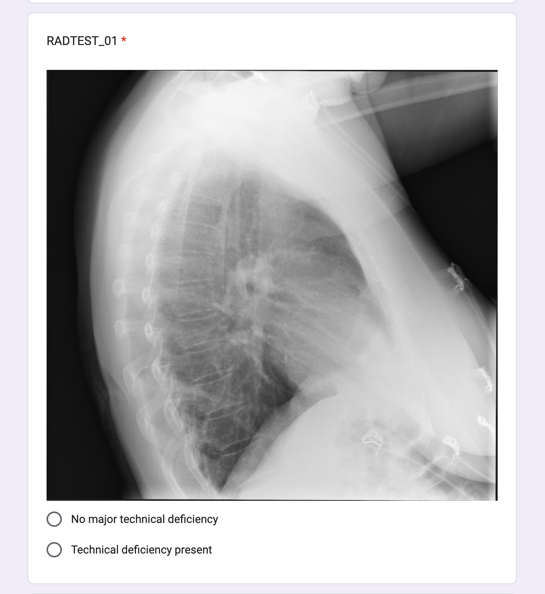


*Figure S1 - Screenshot of Google Form survey question provided to radiologists.*

The radiologist survey aims to have professionals identify technical deficiencies in X-ray images. The goal is to compare the assessment of professionals with that of trained artificial intelligence models to ensure consistency and accuracy in identifying these deficiencies. Training this model aims to provide feedback through auditing chest X-ray datasets and to help guide staff members' decision-making on repeating an X-ray. Future works could explore a similar model at the console following acquisition to flag images that may require a repeat.

*
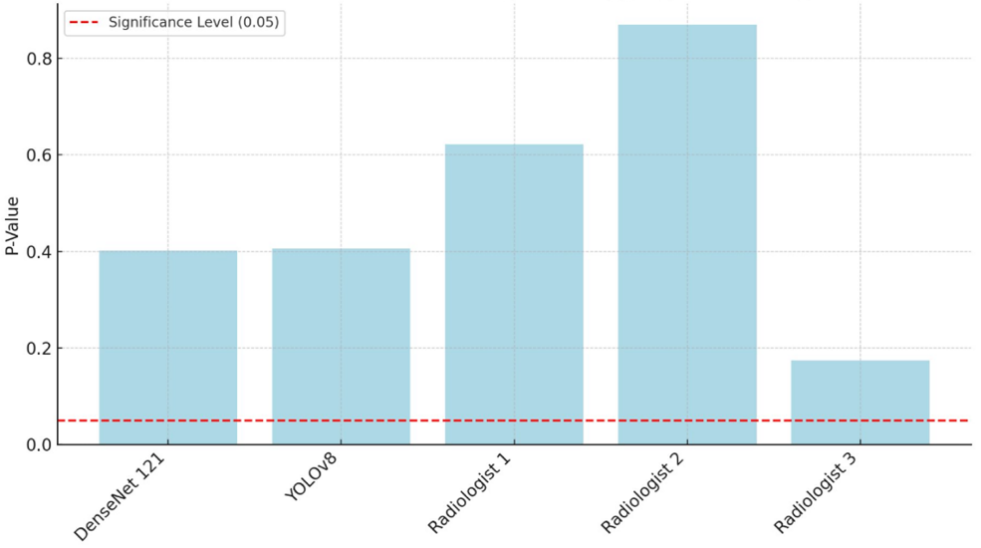
*

*Figure S2 - Chi-squared analysis comparing the classification performance of DenseNet121 and YOLOv8 with that of individual radiologists.*

This figure reflects the aggregation of classification outcomes from all three radiologists into a single, combined confusion matrix. By consolidating their assessments, we created a consensus-based reference that reduces variability from individual judgment and serves as a benchmark for comparison. This combined matrix was used in the chi-squared statistical test to evaluate whether there were significant differences in classification outcomes between the AI models and human experts. The analysis demonstrated that, while minor differences existed, the models performed comparably to radiologists overall — with no statistically significant difference, supporting their potential use in clinical quality assurance workflow.

*\*

Table S1 outlines the technical standards used to assess the diagnostic quality of chest radiographs (CXRs), specifically for posteroanterior (PA) and lateral projections. These criteria are adopted from established guidelines such as the European Commission on Radiographic Quality, and are commonly used by radiologists and radiographers to determine whether a CXR is acceptable or should be repeated.

*Table S1- Image quality criteria for chest radiographs^25^*

| Projections | PA | Lateral |
| --- | --- | --- |
| Inspiration | Deep inspiration is illustrated by the position of ribs above the diaphragm (6 anteriorly or 10 posteriorly) | Full inspiration with suspended respiration |
| Rotation | Spinous process positioned central between the medial ends of the clavicles, illustrating symmetrical thorax | Superimposition of posterior lung borders |
| Anatomy inclusion | Lung apices to costophrenic angles | Lung apices to costophrenic angles |
| Positioning | Medial scapular border is outside of lung fields | Arms raised and are clear of thorax |
| Image quality | Sharp vascular pattern in the entire lung  Clear visualisation of (a) trachea and proximal bronchi, (b) heart and aortic borders, (c) diaphragm and lateral costophrenic angles  Visualisation of spine through heart shadow, retrocardiac lung and mediastinum | Clear visualisation of the diaphragm, mediastinum, posterior border of heart, aorta, mediastinum, sternum, and thoracic spine. |
