# Supplementary material for: Using Convolutional Neural Networks for the Classification of Suboptimal Chest Radiographs
Source: J Med Radiat Sci. 2025 Jul 18;73(1):60–8. doi: 10.1002/jmrs.70006 (PMC12950515; doi:10.1002/jmrs.70006)
Supplement: Supplementary file 2 — Appendix S2. [file JMRS-73-60-s002.docx]

**Appendix 2**

| Term | Definition |
| --- | --- |
| Convolutional neural networks (CNNs) | Specialised deep learning applications engineered to independently learn, extract, and categorise complex features present in images^2^. |
| Hyperparameters | Machine learning variables that regulate the learning process of the model and establish its architecture^22^. |
| Epoch number | Epoch number refers to the number of times a machine learning model has gone through the entire training dataset^21^. |
| Learning rate | It determines the step size at which the model updates its parameters during training. |
| Overfitting | Overfitting transpires when a machine learning model assimilates the intricacies and noise of the training data to such a degree that it detrimentally affects the model's efficacy on new data. In healthcare, overfitting may result in inaccurate predictions and diagnoses. |
| Training/validation loss | Training/validation loss is a metric in machine learning model development. It measures the error between the predicted output and the actual target during the training/validation phase. A lower training/validation loss indicates that the model is learning to make accurate predictions. |
